# Enhanced antibacterial properties of biocompatible titanium *via* electrochemically deposited Ag/TiO_2_ nanotubes and chitosan–gelatin–Ag–ZnO complex coating

**DOI:** 10.1039/c8ra07682k

**Published:** 2019-02-05

**Authors:** Linling Yin, Zhenxuan Fu, Yuan Li, Bin Liu, Zongjian Lin, Jiayu Lu, Xu Chen, Xiaopeng Han, Yida Deng, Wenbin Hu, Derong Zou, Cheng Zhong

**Affiliations:** Department of Stomatology, Shanghai Jiao Tong University Affiliated Sixth People's Hospital Shanghai 310000 China drzou@sjtu.edu.cn; Key Laboratory of Advanced Ceramics and Machining Technology (Ministry of Education), Tianjin Key Laboratory of Composite and Functional Materials, School of Materials Science and Engineering, Tianjin University Tianjin 300072 China cheng.zhong@tju.edu.cn; State Key Laboratory of Metal Matrix Composites, Shanghai Jiao Tong University Shanghai 200240 China

## Abstract

A novel double-layered antibacterial coating was fabricated on pure titanium (Ti) *via* a simple three-step electrodeposition process. Scanning electronic microscopy (SEM) images show that the coating was constructed with the inner layer of TiO_2_ nanotubes doped with silver nanoparticles (TNTs/Ag) and the outer layer of chitosan–gelatin mixture with zinc oxide and silver nanoparticles (CS–Gel–Ag–ZnO). In comparison, we also investigated the composition, structure and antibacterial properties of pure Ti coated with TNTs, TNTs/Ag or TNTs/Ag + CS–Gel–Ag–ZnO, respectively. The TNTs was about 100 nm wide and 240 nm to 370 nm tall, and most Ag nanoparticles (Ag NPs) with diameter smaller than 20 nm were successfully deposited inside the tubes. The CS–Gel–Ag–ZnO layer was continuous and uniform. Antibacterial activity against planktonic and adherent bacteria were both investigated. Agar diffusion test against *Staphylococcus aureus* (*S. aureus*) shows improved antibacterial capacity of the TNTs/Ag + CS–Gel–Ag–ZnO coating, with a clear zone of inhibition (ZOI) up to 14.5 mm wide. Dead adherent bacteria were found on the surface by SEM. The antibacterial rate against planktonic *S. aureus* was as high as 99.2% over the 24 h incubation period.

## Abstract 2

A novel complex antibacterial coating fabricated *via* a simple three-step electrodeposition process shows high antibacterial rate of 99.2%.

## Graphical abstract

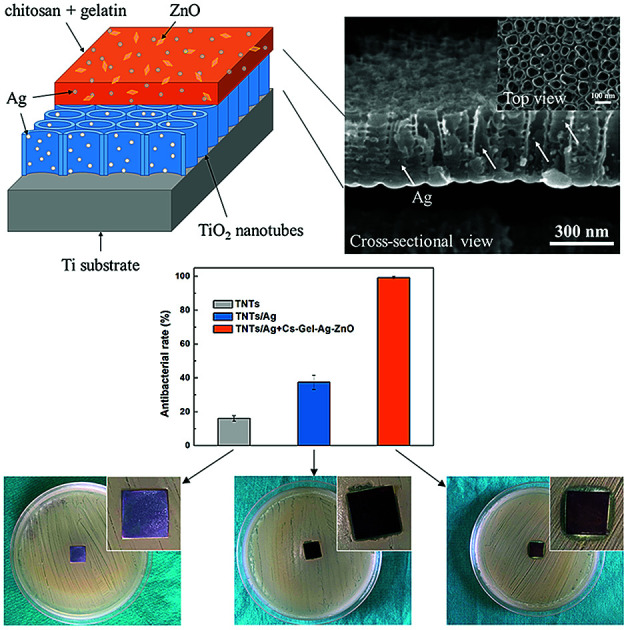

## 1. Introduction

Titanium (Ti) and its alloys as biomedical materials have gained much attention over recent decades due to their superior biocompatibility as well as low cytotoxicity, high wear and corrosion resistance, and high fatigue strength.^1^ More importantly, the relatively low modulus of elasticity compared with biomedical stainless steels and cobalt-based alloys could effectively reduce the stress shielding caused by the mismatch in modulus between bone and implant.^2–4^ Therefore, Ti and its alloys are primarily used as orthopedic and dental implants.^5–10^ Besides, the relative inactivity of Ti in human body environment and its capability of osseointegration make Ti-based materials possible for long-term implantation.^1,4,11–14^ Unfortunately, such biocompatibility also gives rise to bacterial adhesion which could cause severe post-surgical infections.^15^

Many surface modification techniques have been promoted to enhance the antibacterial property of Ti and its alloys.^14–21^ According to the antibacterial agent and mechanism, these techniques can be categorized as: antibiotic modification, non-antibiotic antimicrobial modification, and anti-adhesion modification.^20^ Among the many non-antibiotic antimicrobial agents, silver (Ag) is believed to be one of the strongest with a broad spectrum of antibacterial activities.^22–27^ Depending on the modification process, Ag can be introduced as either metallic or ionic state.^28^ Silver nanoparticles (Ag NPs) outreach other existing form of Ag in that the extremely large surface area and small particle size render them highly reactive with proteins and DNAs and easy to penetrate the cell wall of bacteria.^26^ However, silver nanoparticles are reported to be cytotoxic due to the burst release of Ag ions.^15^

A common approach is to load Ag NPs onto Ti which has a modified surface structure. As it is difficult for body fluids to infiltrate into the complex structure of the host, slow Ag ions release and consequently controlled cytotoxicity are expected. In our previous work,^29^ TiO_2_ nanotubes (TNTs) on Ti foil was successfully prepared through electrochemical deposition process. One advantage of such structure is that vertically aligned TNTs are of considerably high surface area,^30,31^ where the Ag NPs are more dispersive than on flat surfaces. As a result, Ag NPs are potentially highly available whilst keeping a long-lasting antibacterial activity. However, the release rate of Ag ions in TNTs/Ag is not efficiently high due to the complex structure of TNTs, limiting the antibacterial performance of the coating. Amin Yavari *et al.*^32^ developed a porous Ti implant coated with Ag-doped TNTs. The Ag ion concentration during *in vitro* test dropped sharply after 3 days. Consequently, antibacterial rates against planktonic and adherent bacteria both dropped. In addition, Zhao *et al.*^33^ prepared Ag NPs loaded TNTs with different diameters *via* anodization and heat treatment followed by photo-reduction. The antibacterial rate against *S. aureus* after 24 h incubation was up to 82.9% (in the dark) demonstrating a limited antibacterial rate.

Another approach is to dope Ag NPs in polymer matrix to form continuous coatings. Chitosan (CS) is a kind of antimicrobial polymers that is frequently employed as scaffold and implant coating material.^34^ For instance, Li *et al.*^15^ fabricated CS/Ag complex coatings on NiTi alloy *via* electrochemical codeposition. They compared the antibacterial properties of Ag, CS and CS/Ag coated Ti and found that antibacterial rates of Ag and CS were very close, whereas the CS/Ag coating exhibited strongest antibacterial activity due to the highest release rate of Ag ions. CS is compatible with human cells due to its molecule structure similar to those of hyaluronic acid and glycosaminoglycan extracellular matrix molecules.^12^ Regiel *et al.*^21^ studied the influence of CS on the formation of Ag NPs. They found that a high deacetylation degree could help nucleation and prevent agglomeration so as to form small and dispersed Ag NPs. In addition, CS is pH-dependent soluble so as to keep an efficient release rate of Ag NPs or Ag ions. Given the intrinsic biocompatibility and antibacterial nature of CS, it is hence desirable to incorporate Ag NPs into CS to enhance the antibacterial property whereas reducing the cytotoxicity of Ag NPs and retaining biocompatibility.

Compared with CS/Ag coating, although the TNTs/Ag coating is possible to suffer from limited biocompatibility for the higher exposure of reactive Ag-NPs, it is remarkable for a larger Ag capacity. Therefore, in the present work, we propose a novel hierarchical structure combining TNTs and the biocompatible CS to ensure both the durability and biocompatibility. The composite structure and the complex coatings in our work provide a combination of several advantages. First, the combined coating was fabricated on pure Ti *via* a simple three-step electrodeposition process, where in the last step CS + Gel + Ag-NPs + ZnO-NPs are co-electrodeposited onto TNTs/Ag layer. The electrochemical deposition as an *in situ* formation technique is especially suitable for biomedical implants due to the good adherence and high purity at the coating/substrate interface. Second, in the present work, Ag nanoparticles were grown inside TNTs by simple electrodeposition method with the size of less than 20 nm. Third, the complex coatings in our work are combined with several compositions with antibacterial activity and can play the synergistic role for further enhancing the activity. Gelatin (Gel) is added into the CS-based composites for rigid adhesion between CS and TNTs. To further increase the antibacterial activity of the coatings, ZnO nanoparticles, another strong non-toxic bactericide^35^ is introduced into the chitosan matrix. It was found that ZnO and Ag could mutually enhance the antimicrobial activities through synergistic effect.^36^ In addition, ZnO nanoparticles are more effective against Gram-positive bacteria like *S. aureus* than Gram-negative ones, such as *Escherichia coli*, whereas Ag nanoparticles are the opposite,^35^ which makes the bactericidal effect of Ag/ZnO nanohybrid even enhanced. Due to the intrinsic antibacterial activity against planktonic bacteria of the CS, ZnO and Ag NPs, and the synergistic effect between ZnO and Ag, the novel composite coating in our work reaches a significantly high antibacterial rate of 99.2%, which shows the promising applications in the fields of orthopedic and dental implants. The Ti substrates coated with anodic TNTs/Ag complex, and TNTs/Ag + CS–Gel–Ag–ZnO were separately prepared and characterized by scanning electron microscopy (SEM), energy dispersive X-ray (EDX) spectroscopy, X-ray diffraction (XRD), and X-ray photoelectron spectroscopy (XPS). The adhesion strengths of the coatings were investigated by cross-cut tape test. The *in vitro* antibacterial properties of the four samples (including one bare Ti as the control) were investigated by agar diffusion method and plate counting method, and their results are presented in the form of inhibition zones, SEM micrographs and antibacterial rates.

## 2. Experimental

### 2.1 Sample preparation

#### 2.1.1 Pretreatment of Ti plates

The as-received pure Ti sheets (Ti wt% > 99.9%), with the specific size of 10 mm × 10 mm × 0.25 mm, were first ground by SiC paper with gird of #1200 and carefully rinsed with deionized water. The polished Ti sheets were ultrasonically cleaned using acetone for 30 minutes in order to remove organic adherents. Then they were ultrasonically cleaned in deionized water. After another 30 minutes, these Ti sheets were immersed into acid (HF : HNO_3_ = 1 : 3 vol) for a few seconds to remove the oxidized layer.

#### 2.1.2 Anodization of Ti (preparation of TNTs)

An as-prepared aqueous solution with the mixture of 0.1 M NaF and 0.5 M NaHSO_4_ was adopted as the electrolyte for anodization. The anodization process was carried out in a conventional two-electrode system, powered by KXN-6010D. A Ti sheet acted as the anode, and the cathode was a graphite plate. The distance of the electrodes was set to 2 cm. Anodization took 30 minutes, under constant voltage of 15 V dc.

#### 2.1.3 Electrodeposition of Ag NPs

A mixed solution of 10 mM AgNO_3_ and 100 mM NaNO_3_ was used as the electrolyte solution. The previously prepared anodic Ti sheets were used as the working electrode, and the graphite plate was the counter electrode. A pulse current of 10 mA cm^−2^ ac was applied at the interval of 0.3 s and the holding time was 0.1 s. After 100 cycles, the prepared sample was rinsed with deionized water.

#### 2.1.4 Fabrication of CS–Gel–Ag–ZnO coatings

First, 0.625 mL of glacial acetic acid was dissolved in 100 mL of deionized water, followed by dissolving 0.5 g of chitosan powder and the solution pH was adjusted with 1 mL NaOH solution (6 M). Then, 0.25 g of gelatin was dissolved in the above solution at 40 °C. Under magnetic stirring for 1 h, zinc nitrate (25 mM), silver nitrate (1 mM), and sodium nitrate (0.0925 M) were added to the above solution, thus obtaining the electrolyte. The sample prepared in the third step was used as the cathode, and the graphite plate was used as the anode (the distance was 2 cm) at a constant current voltage of 3 V for 60 seconds, and then rinsed with deionized water and dried in air. The CS–Gel–Ag, CS–Gel–ZnO, CS–Gel coatings were fabricated by the same method as CS–Gel–Ag–ZnO.

### 2.2 Characterization

#### 2.2.1 Scanning electron microscopy (SEM)

We use scanning electron microscopy (SEM, FEI QuantaTM 250 FEG, USA) to characterize the top-surface and cross-section morphologies. For top-surfaces, the sample sheets were fixed on the stages by conductive adhesive, with the coated side face up. For cross-sections of TNTs and TNTs/Ag, the samples were first immersed in liquid nitrogen (N_2_) for 5 min, and then fractured immediately after taken out. The fractured samples were pasted on a convex stage with their cross-section face up.

#### 2.2.2 Energy dispersive X-ray spectrometry (EDS)

The surface composition was characterized by energy dispersive X-ray spectrometry (EDS) incorporated in the SEM.

#### 2.2.3 X-ray diffraction (XRD)

X-ray diffraction (XRD, D8 Advanced, Brüker, US) with Cu Kα radiation was employed to determine the phase constituent of the coating. The supply voltage was 40 kV and the tube current was 40 mA, scan starting from 5° to 80°.

#### 2.2.4 X-ray photoelectron spectrometry (XPS)

Valence states of elements on the surface were determined by X-ray photoelectron spectroscopy (XPS, escalab 250Xi, Thermo Fisher, US) with Al Kα radiation.

#### 2.2.5 Cross-cut tape test

The adhesion strengths of the coatings to the substrate were measured qualitatively using ASTM D 3359-02: Standard Test Methods for Measuring Adhesion by Tape; cross-cut tape test (B). In the center of the coated side on the 10 mm × 10 mm sample, a 6 × 6 grid was made using parallel cuts down to the substrate with approximately 1 mm gaps between each cut. An adhesive tape (Scotch Tape, 3M) was firmly attached on to the area of the grid for 90 s and was then pulled abruptly off at an angle as close to 180° as possible. Damage of the coating was evaluated visually. The adhesion strength of the coating was classified into 5 scales according to the percentage of damaged area: 0% (5B), less than 5% (4B), 5–15% (3B), 15–35% (2B), 35–65% (1B), over 65% (0B).

#### 2.2.6 Agar diffusion test

Agar diffusion tests were performed against *Staphylococcus aureus* (*S. aureus*, ATCC29213). At first 10 μL *S. aureus*, which has been adjusted to 1.5 × 10^8^ CFU mL^−1^, was spread evenly over Mueller–Hinton plates. Sample Ti foils were separately placed in the center of the prepared plates with their coated face down. After incubation at 37 °C for 24 h, the plates were shot by digital camera. All the samples were tested for at least three times.

#### 2.2.7 Adherent bacteria examination

The antibacterial ability of the samples against adherent bacteria was qualitatively tested by scanning electron microscopy (SEM; FEI QuantaTM 250 FEG, USA). The samples were immersed in the suspension of *S. aureus* with the concentration of 1.5 × 10^8^ CFU mL^−1^. After incubation at 37 °C for 24 h, the samples were rinsed with phosphate buffer saline (PBS) in order to remove non-adherent bacteria. The adherent bacteria on the samples were fixed with 2.5 wt% glutaraldehyde solution at 4 °C for 4 h. Then they were dehydrated in ethanol subsequently from 30 vol%, 50 vol%, 70 vol%, 90 vol% to 100 vol%. Ethanol was replaced by amyl acetate in an amyl acetate solution at room temperature for 1 h. After that, the samples were dried at critical point, and then sputtered with Au for SEM imaging.

#### 2.2.8 Antibacterial rate assay

The antibacterial activity against planktonic bacteria was tested in a trypticase soy broth (TSB) medium at 37 °C containing *S. aureus*, which was adjusted to the concentration of 1.5 × 10^8^ CFU mL^−1^. The samples were incubated in 1 mL such suspension for 24 h. A pure Ti foil was also tested as the control. Then the suspension was collected to count viable planktonic bacteria using serial dilution and spread-plate method. The antibacterial activity against planktonic bacteria was represented by the antibacterial ratio (*R*_a_, %), following:1
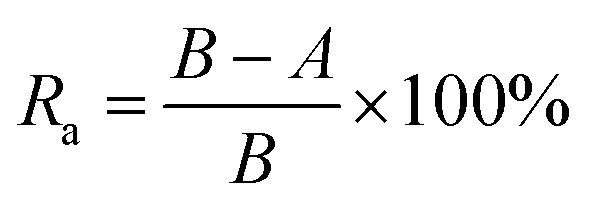
where *A* is the average number of bacteria on the tested sample, *B* is the average number of bacteria on the control sample. Each experiment was tested for five times. The results are presented in the form of means ± standard deviations (SD).

## 3. Results and discussion

### 3.1 Surface morphology and structural characterizations

#### 3.1.1 TNTs

**Fig. 1:**
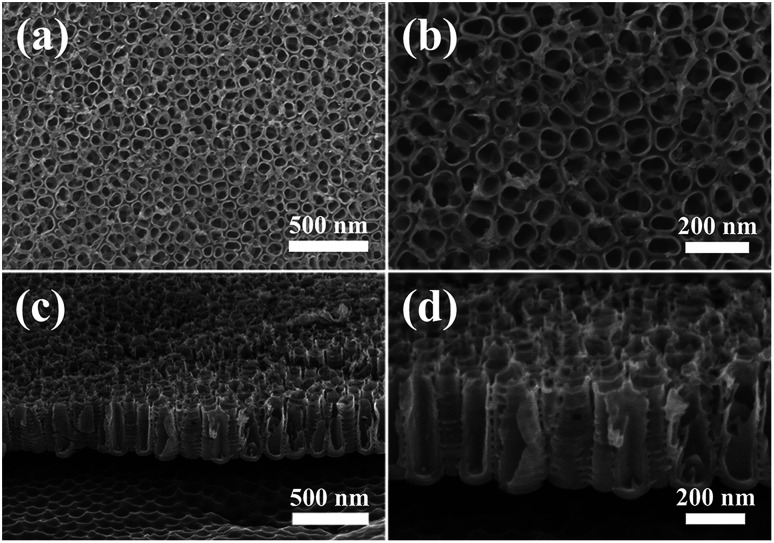
SEM micrographs of the TNTs on Ti substrate: surface morphology with (a) low and (b) high magnification, and cross-sectional morphology of TNTs with (c) low and (d) high magnification.

The surface topography and cross-sectional images of the Ti sample after anodic oxidation are characterized by SEM, and the results are given in Fig. 1. Vertically aligned and compactly arranged TiO_2_ tubes with open tops are formed on the Ti substrate. It is obvious that the most severe corrosion happens inside the tubes and the areas surrounded by the tubes are less etched, whilst the walls undergo slightest electrochemical corrosion (Fig. 1b). These tubes are randomly distributed where there is no detective aggregation. Most of them have roughly circular sectional profiles, whose diameters are mostly around 80 to 100 nm. The center-to-center distance of TNTs is about 100 nm and the wall thickness about only 20 nm, indicating high surface porosity which makes it easier for Ag NPs to deposit inside TNTs. The height of as-synthesized TNTs ranges from 240 nm to 370 nm, and their walls have corrugated exterior, which can be observed from their cross-sectional SEM micrographs (Fig. 1c and d).

#### 3.1.2 Ag-loaded TNTs

**Fig. 2:**
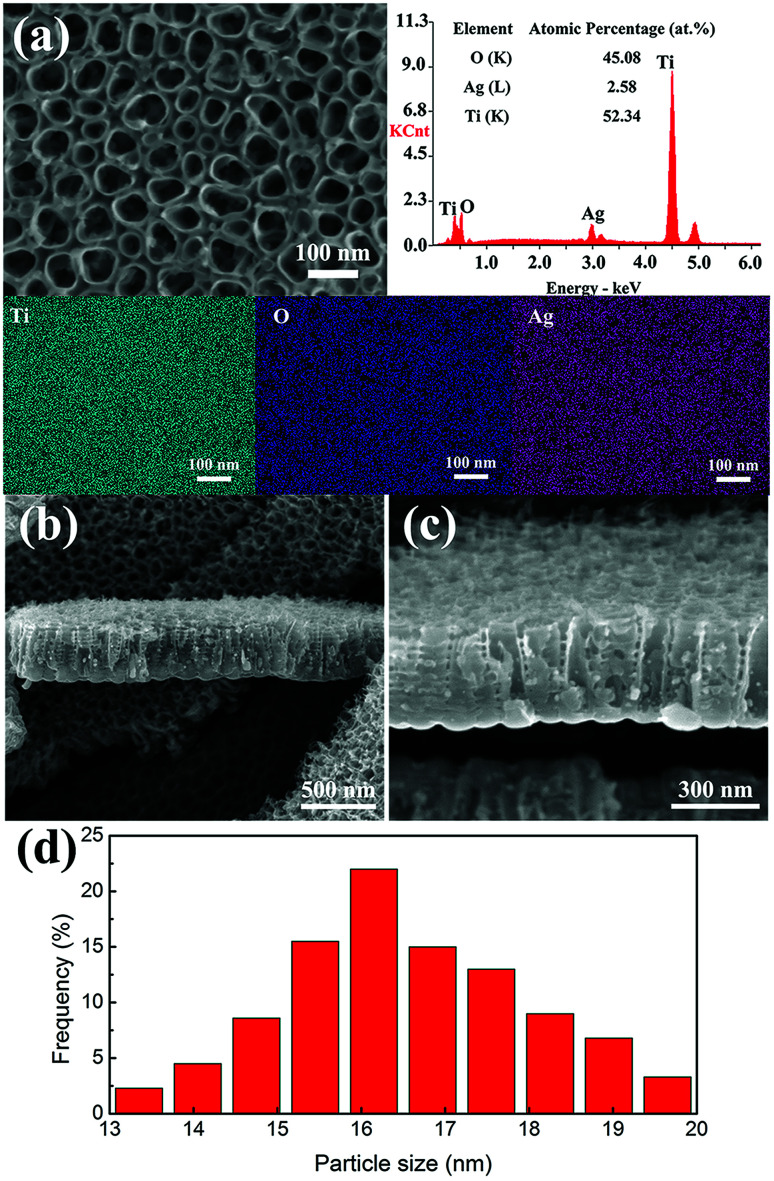
SEM micrographs of the TNTs loaded with Ag NPs: (a) surface morphology from top view and corresponding EDS results where quantitative composition data are enclosed, and cross-sectional morphology with (b) low and (c) high magnifications and (d) corresponding Ag nanoparticle size distribution histogram.

The SEM images of the top surface morphology of the TNTs after electrodeposition of Ag and the corresponding EDS results are given in Fig. 2a–c show the cross-sectional morphology. Only few particles (light spots in Fig. 2a) are found on the top surface, while the EDS mapping indicates uniform Ag distribution all over the surface. The size distribution histogram of Ag nanoparticles is shown in Fig. 2d, showing that most Ag nanoparticles exhibit particle size of no more than 20 nm, which is much smaller than the diameter of the TNTs. The EDS point spectrum also clearly suggests the presence of Ag, indicating the successful formation of Ag nanoparticles inside the TNTs. Consequently, TNTs after Ag electrodeposition still remain open, which ensures effective mass exchange between the inner Ag NPs and the outer environment. It can be seen from the cross-sectional images of TNTs (Fig. 2b and c) that the structure of TNTs is not changed or destroyed and that some Ag NPs are deposited onto the inner wall and bottom of TNTs, which has been rarely reported in previous researches. Xie *et al.*^37^ reported Ag NPs loaded TNTs *via* pulse current deposition (PCD). Most of the Ag NPs were deposited on the top of TNTs, with only few at the bottom, partly due to the uneven distributional current density during the PCD. Later, Zhang *et al.*^38^ prepared the same structure *via* direct current electrodeposition. Few Ag NPs were found inside the tubes, and the aggregation of Ag NPs could not be prevented once extending the deposition time.

#### 3.1.3 TNTs/Ag + CS–Gel–Ag–ZnO

**Fig. 3:**
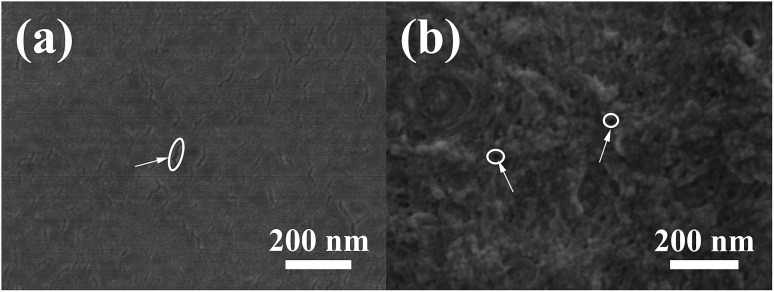
SEM micrographs of TNTs/Ag coated (a) with CS–Gel and (b) with TNTs/Ag + CS–Gel–Ag–ZnO composite.

The SEM images of TNTs/Ag + CS–Gel–Ag–ZnO composite coating are shown as Fig. 3b. CS–Gel coating on TNTs/Ag without electrodeposition of Ag and ZnO is also provided for comparison (Fig. 3a). The compact, continuous and homogenous CS–Gel coating exhibits no complicated micro or nano-structural characteristics, while there only exist some small grooves on the surface (indicated by the circle along with white arrow). Furthermore, dark spots are dimly visible on the CS–Gel surface. Considering the top surface morphology of TNTs in SEM graph at the same magnification, as shown in Fig. 1a, the spot pattern should be caused by microscopic unevenness correspondent to the underneath tubes. On the other hand, the surface of TNTs/Ag + CS–Gel–Ag–ZnO coating is marbled with wrinkles in the SEM graphs (Fig. 3b), which may be the pattern of co-deposited ZnO and Ag. Small holes with tens of nanometers size are found all over the surface (indicated by the circles along with white arrows). A possible explanation is that these holes were left by the re-dissolved ZnO or Ag particles during the electrodeposition process. Given the antibacterial activity of TNTs/Ag, the porous structure of CS–Gel–Ag–ZnO coating is an advantage, which allows for sustained release of Ag NPs from the underneath TNTs to the environment.

**Fig. 4:**
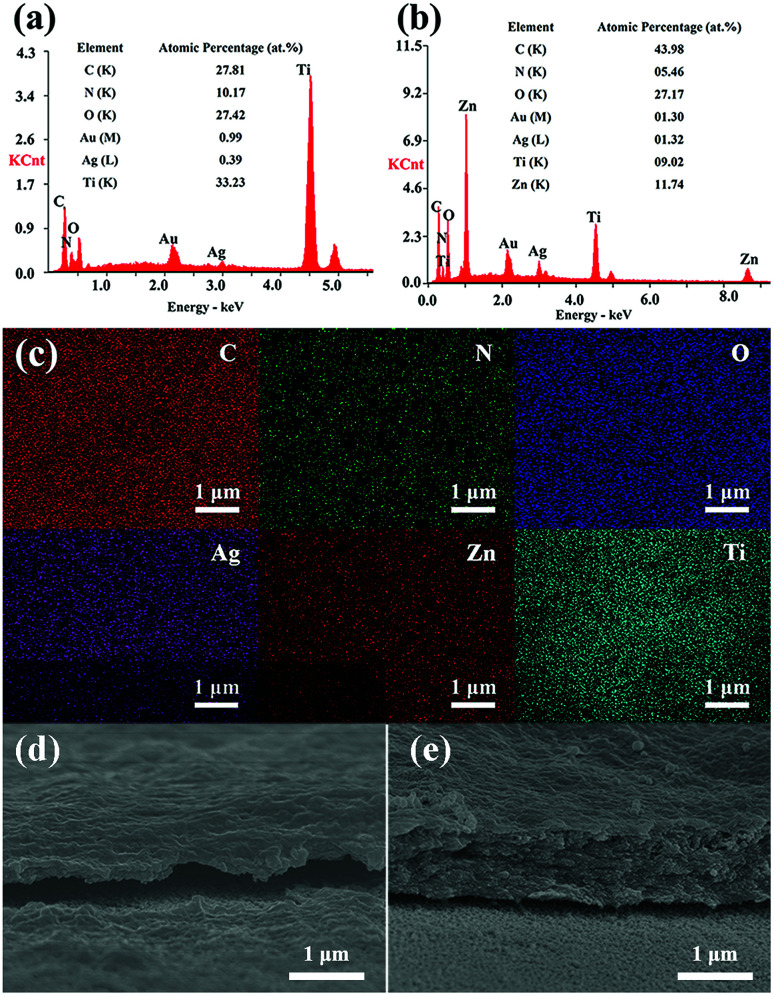
EDS point scanning spectra with quantitative composition data for the (a) TNTs/Ag + CS–Gel and (b) TNTs/Ag + CS–Gel–Ag–ZnO complex coatings and (c) corresponding EDS mappings for the latter of the area of interest and cross-sectional SEM images of (d) TNTs/Ag + CS–Gel and (e) TNTs/Ag + CS–Gel–Ag–ZnO.

Fig. 4 shows the EDS results of TNTs/Ag + CS–Gel and TNTs/Ag + CS–Gel–Ag–ZnO coatings. In Fig. 4a and b, the gold (Au) content come from the sprayed Au in preparation of samples for SEM observation. In comparison to the TNTs/Ag coating, carbon (C) and nitrogen (N) elements are found in the two complex coatings, in accordance with the existence of CS and Gel. The C and N contents in the results obtained by EDS method were just for qualitatively analysing since EDS measurements have the limitation for quantitative determination of the light elements.^39,40^ Ti is still found in the two coatings since the stimulated X-ray used for EDS measurement came from the area up to several micrometers deep. It can be seen in Fig. 4d and e, both the thickness of the layer of TNTs/Ag + CS–Gel and TNTs/Ag + CS–Gel–Ag–ZnO is less than 2 μm, which is thin enough for the detection of X-ray from the Ti element. The Ag–ZnO free coating where the pre-deposited Ag in TNTs should be the only source of Ag exhibited lowest Ag contents (only 0.39 at%), whilst the Ag–ZnO containing coating contained 1.32 at% Ag, still lower than the 2.58 at% Ag content in the TNTs/Ag coating (Fig. 2a). In addition, Zn occurred in the TNTs/Ag + CS–Gel–Ag–ZnO coating and its content was about 11.74 at%. For further investigating the distribution of elements on the TNTs/Ag + CS–Gel–Ag–ZnO coating, its EDS mapping images were given in Fig. 4c. The EDS mapping images exhibit uniformly distributed C, N, O, Zn and Ag, which indicates that ZnO and Ag NPs were highly dispersive throughout the whole surface.

Besides, to analyse the contents of Ag and Zn in the TNTs/Ag + CS–Gel–Ag–ZnO complex coating, XPS measurements were carried out. According to the results in Table 1, the contents of Ag, Zn, C, N, and O in the coating are 1.91 at%, 10.93 at%, 55.47 at%, 10.31 at%, and 21.38 at%, respectively. In addition, the C, N and O element contents of TNTs/Ag + CS–Gel from XPS are 72.38 at%, 13.52 at%, and 14.10 at%, respectively. Compared with TNTs/Ag + CS–Gel, the TNTs/Ag + CS–Gel–Ag–ZnO coating exhibits additional Ag and Zn contents, which demonstrates that Ag and Zn have been successfully co-deposited with CS and Gel.

**Table 1:** Atomic percentages from XPS measurement of TNTs/Ag + CS–Gel–Ag–ZnO and TNTs/Ag + CS–Gel

Samples	Ag (at%)	Zn (at%)	C (at%)	N (at%)	O (at%)
TNTs/Ag + CS–Gel–Ag–ZnO	1.91	10.93	55.47	10.31	21.38
TNTs/Ag + CS–Gel	—	—	72.38	13.52	14.10

**Fig. 5:**
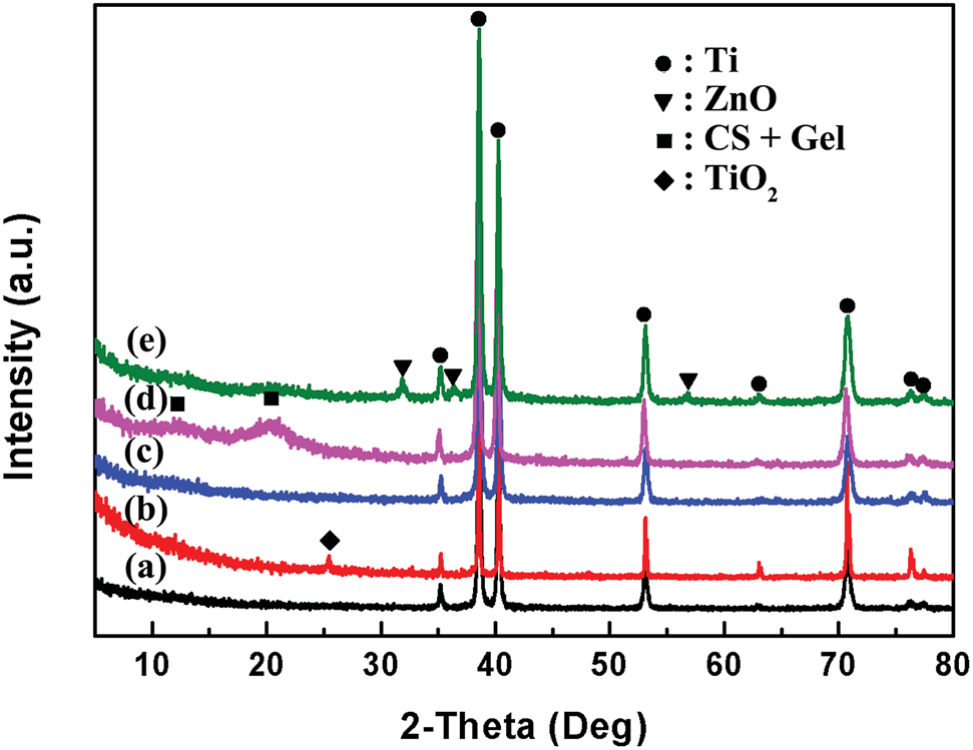
XRD spectra of (a) Ti, (b) TNTs coating after calcination at 450 °C, (c) TNTs/Ag coating, (d) TNTs/Ag + CS–Gel and (e) TNTs/Ag + CS–Gel– Ag–ZnO complex coatings.

The XRD spectra of bare and coated Ti samples are shown in Fig. 5. The peaks of the bare Ti (marked black) are coincident with the XRD pattern of α-Ti, with the first three characteristic peaks at 2*θ* = 35.2°, 38.5° and 40.2°. The position and intensity of substrate Ti peaks remain stable at coated samples. A new peak at about 2*θ* = 25.4° occurs in the spectrum of calcinated Ti (marked red), which is attributed to anatase TiO_2_. Freshly deposited TNTs *via* anodization process are amorphous with no peaks in the XRD results. After calcinated at 450 °C in nitrogen atmosphere, the amorphous TiO_2_ converts to crystallize TiO_2_ with the peak at about 2*θ* = 25.4°, indicating that the TNTs are successfully formed by the simple electrodeposition process. There are two broad peaks at 2*θ* = 11.8° and 20.4° in the XRD pattern of TNTs/Ag + CS–Gel coating (marked pink), corresponding to the semi-crystalline nature of chitosan. The peaks are wider when compared to the XRD pattern of pure chitosan, which should be caused by the presence non-crystalline gelatin that reduced the crystallinity of the blending. ZnO were found in the TNTs/Ag + CS–Gel–Ag–ZnO coating for the unique peaks occurring at around 2*θ* = 31.9°, 36.3° and 56.6° in the spectrum (marked green). On the other hand, the characteristic peaks of Ag were found neither in the TNTs/Ag or TNTs/Ag + CS–Gel–Ag–ZnO coated samples. We suppose that the Ag content was too scarce to be detected by XRD, though it was detectable for EDS.

**Fig. 6:**
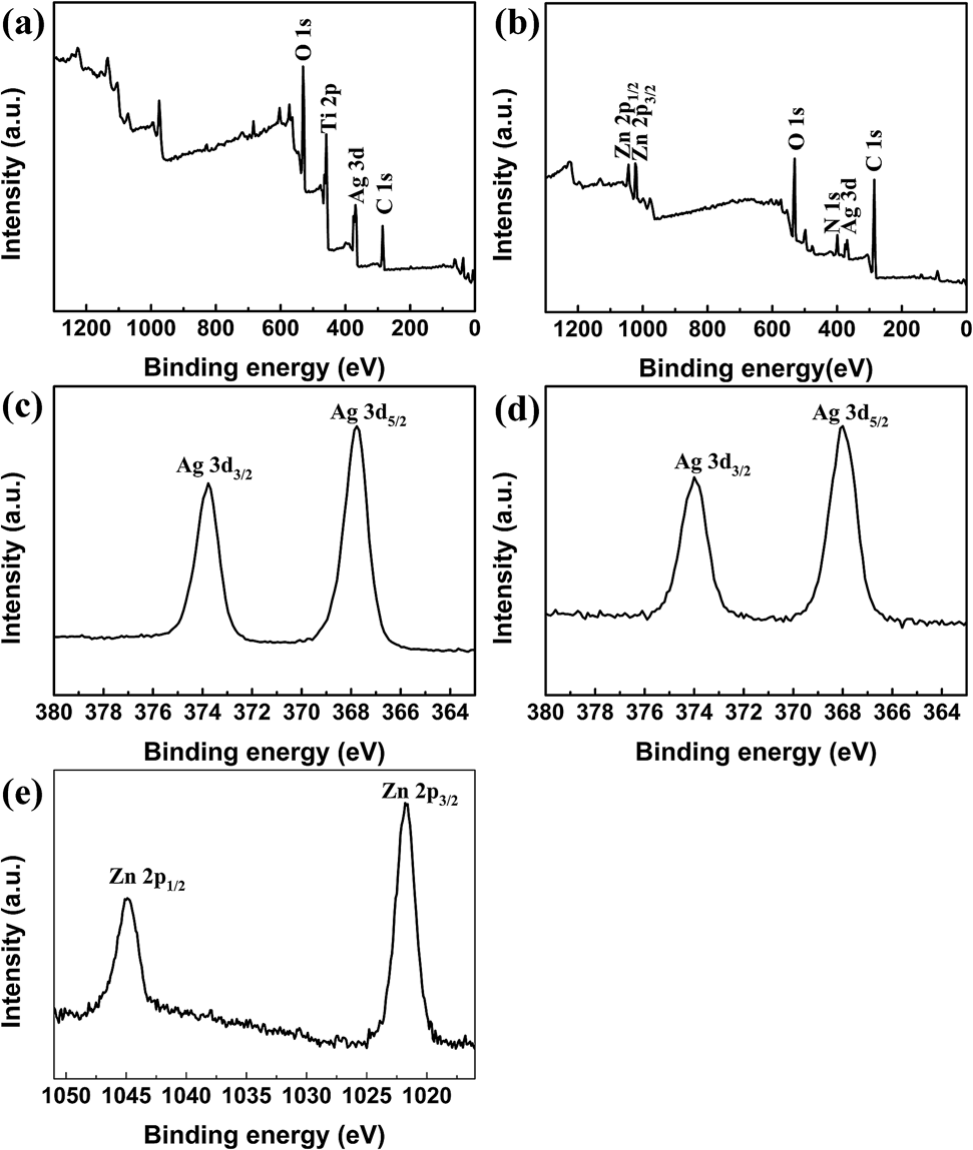
XPS spectra of Ti samples coated with (a) TNTs/Ag and (b) TNTs/Ag + CS–Gel–Ag–ZnO and narrow scans for (c) Ag 3d peaks of TNTs/Ag and (d) Ag 3d and (e) Zn 2p peaks of TNTs/Ag + CS–Gel–Ag–ZnO coating.

XPS spectra of TNTs/Ag on Ti samples with and without CS–Gel–Ag–ZnO coating were provided as Fig. 6 XPS is a semi-quantitative tool frequently used for characterizing the species and chemical states of elements on the surface. Fig. 6a and c show full-scale scans for the two samples from 0 to 1300 eV binding energy. The peaks of O 1s, Ti 2p and Ag 3d occurred in the spectrum of TNTs/Ag, and peaks of Zn 2p, O 1s, N 1s, Ag 3d were detected in the spectrum of TNTs/Ag + CS–Gel–Ag–ZnO. For the TNTs/Ag + CS–Gel–Ag–ZnO, it is obvious that the signal of Ti disappeared and a new N signal corresponding to CS occurred. Therefore, the TNTs/Ag layer was successfully covered by the CS–Gel–Ag–ZnO coating, which was continuous and undestroyed. Fig. 6c shows the narrow scan of Ag 3d peaks of TNTs/Ag. The Ag 3d_5/2_ peak occurred at 367.8 eV, and the Ag 3d_3/2_ peak occurred at 373.8 eV, 6.0 eV higher than the d_5/2_ peak. These values highly match the XPS data of metallic Ag, hence indicating merely physical bond of Ag NPs with the TNTs. Fig. 6d shows the narrow scan at Ag peaks of TNTs/Ag + CS–Gel–Ag–ZnO. The 3d_5/2_ peak occurred at 367.99 eV, and the 3d_3/2_ peak occurred at 374 eV. While the peak interval almost equal to the value of TNTs/Ag, the two peaks slightly shifted toward high binding energy, suggesting there were electron transfers between Ag and other electron donors. Thus Ag could be either bonded with ZnO or chelated with CS.^41^ The narrow scan at Zn 2p peaks (Fig. 6e) shows that the Zn 2p_3/2_ was at 1021.74 eV and Zn 2p_1/2_ was at 1044.85 eV, which should be the pattern of ZnO. Therefore, ZnO was successfully co-deposited with CS and Gel.

### 3.2 Adhesion strength

**Fig. 7:**
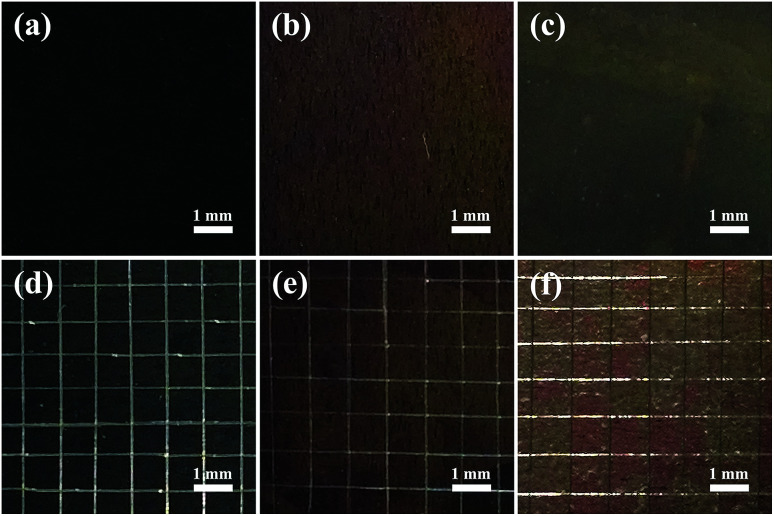
Optical images of the original coatings and the coatings after cross-cut tape test for (a and d) TNTs, (b and e) TNTs/Ag and (c and f) TNTs/Ag + CS–Gel–Ag–ZnO coated Ti samples.

The adhesion strengths of the TNTs, TNTs/Ag and TNTs/Ag + CS–Gel–Ag–ZnO coatings were separately tested by cross-cut tape test. Fig. 7 shows the optical images of the coatings' surfaces before and after the test, and the results are listed in Table 2. After the test, no damages occurred on both the TNTs and TNTs/Ag coatings, indicating excellent adhesion strength between the TNTs and the substrate. This is because TNTs were generated from the substrate during the electrochemical anodization process, and that tube walls are condensed (as Fig. 1c and d show). As we can see from the SEM cross-sectional graphs (Fig. 2b and c), Ag NPs are mostly deposited inside the TNTs, so that the additional Ag NPs will not change the adherence feature of the coating. The TNTs/Ag + CS–Gel–Ag–ZnO coating, however, was not as firm as the other two. The original surface of TNTs/Ag + CS–Gel–Ag–ZnO coated pure Ti, as Fig. 7c shows, was dark brown. After the test, some grids were damaged, and as Fig. 7f shows, their colour slightly turned red. Based on the number of damaged grids, the percentage of damaged area was about 52.8%. According to the ASTM D 3359-02, the adhesion strength of TNTs and TNTs/Ag both reaches 5B, and the adhesion strength of TNTs/Ag + CS–Gel–Ag–ZnO fits in the class of 1B. Considering the microstructure of TNTs, such adherence is probably owing to the large interface area between TNTs and CS–Gel.

**Table 2:** Percentages of damaged area in cross-cut tests and corresponding adhesion strengths

Sample coating	Damaged area (approx. %)	Adhesion strength class
TNTs	0	5B
TNTs/Ag	0	5B
TNTs/Ag + CS–Gel–Ag–ZnO	52.8	1B

### 3.3 *In vitro* antibacterial properties

#### 3.3.1 Agar diffusion test

**Fig. 8:**
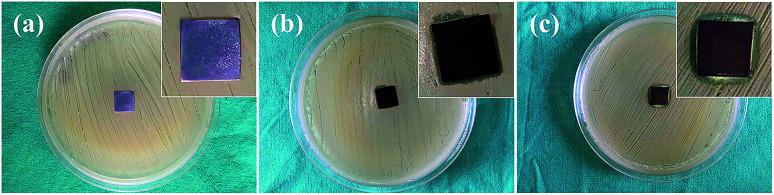
Photographs and close-up images of the ZOI for (a) TNTs, (b) TNTs/Ag and (c) TNTs/Ag + CS–Gel–Ag–ZnO coated Ti samples after agar diffusion test against *S. aureus*.

Agar diffusion test against *S. aureus* was performed on TNTs/Ag + CS–Gel complex coating to qualitatively characterize its antibacterial property. The antibacterial activities of TNTs and TNTs/Ag coatings were also investigated for comparison. The results are shown in Fig. 8. Agar diffusion method is typically used to test the antibiotic sensitivity of bacteria which corresponds to the diameter of the zone of inhibition (ZOI). The ZOI typically occurs during the diffusion of bioactive substances, so that a larger ZOI mainly reflects stronger antibacterial activity against planktonic bacteria. All the three samples were anticipated to be bioactive. However, no ZOI formed in the case of mere TNTs (Fig. 8a), probably owing to the fact that TiO_2_ is a photocatalytic bactericide and that samples were tested in dark. Although ZOI can be observed for the TNTs/Ag sample, it is very small with irregular feature and it is thus difficult to characterize the diameter of the ZOI (Fig. 8b). This is due to its relatively poor antibacterial activity. The slight improvement compared with mere TNTs should be contributed to the Ag NPs inside the TNTs. On the other hand, the TNTs/Ag + CS–Gel–Ag–ZnO coating showed the greatest antibacterial activity in the form of very clear inhibition zone around the sample with a diameter of approximately 14.5 mm (Fig. 8c). The antibacterial activity against planktonic *S. aureus* was significantly enhanced by the bioactive ZnO, Ag and CS contents in the CS–Gel–Ag–ZnO coating.

#### 3.3.2 Microscopic examination of adherent bacteria

**Fig. 9:**
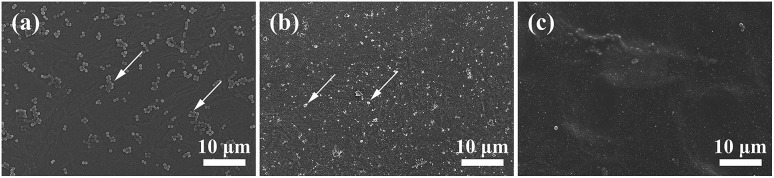
SEM surface micrographs of (a) TNTs, (b) TNTs/Ag and (c) TNTs/Ag + CS–Gel–Ag–ZnO coated samples after 24 h incubation at 2000× magnification.

Adherent bacteria on the surface of the samples were examined by SEM. Fig. 9 shows their SEM surface morphologies. *S. aureus* (dispersedly distributed bright spots) was found on the surface of the three samples, where the TNTs/Ag + CS–Gel–Ag–ZnO coated exhibits fewest bacteria existence. On the surface of TNTs, *S. aureus* with undistorted configuration could be found, while on the surface of TNTs/Ag were fragments of dead *S. aureus*. Therefore, TNTs/Ag coating is capable of killing adherent *S. aureus*. Though dead bacteria were still found on the surface of TNTs/Ag + CS–Gel–Ag–ZnO coating, the amount of fragments was much fewer in comparison with TNTs/Ag, indicating declined concentration of adherent bacteria. Due to the bactericidal activity against planktonic bacteria of TNTs/Ag + CS–Gel–Ag–ZnO as was proved by agar diffusion test, bacteria were killed before they could attach to the surface of the sample. Hence it is reasonable to believe that TNTs/Ag + CS–Gel–Ag–ZnO is capable of killing both planktonic and adherent bacteria, whereas TNTs/Ag could only efficiently kill adherent bacteria but not planktonic. For the TNTs/Ag sample, Ag nanoparticles are localized inside the TNTs and have limited antibacterial activity against planktonic bacteria. On the contrary, for the TNTs/Ag + CS–Gel–Ag–ZnO composite coating, the Ag in the outside composite coating exhibits antibacterial activity against both adherent and planktonic bacteria.

#### 3.3.3 Antibacterial rates (*R*_a_) against planktonic bacteria

**Fig. 10:**
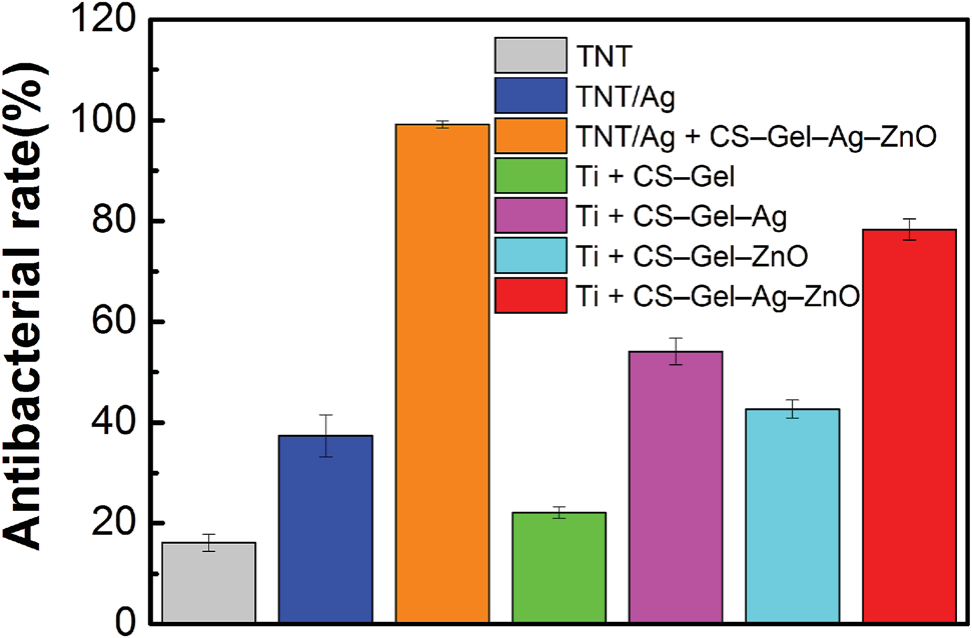
Antibacterial rates of TNTs, TNTs/Ag, TNTs/Ag + CS–Gel–Ag–ZnO, Ti + CS–Gel, Ti + CS–Gel–Ag, Ti + CS–Gel–ZnO, and Ti + CS–Gel–Ag–ZnO coated samples against planktonic *S. aureus* after 24 h incubation. The antibacterial rate data are presented as mean ± SD.

In order to quantify the antibacterial properties, the antibacterial rates (*R*_a_) of TNTs, TNTs/Ag and TNTs/Ag + CS–Gel–Ag–ZnO against planktonic *S. aureus* are presented in Fig. 10. The TNTs/Ag + CS–Gel–Ag–ZnO coating exhibits an impressively high *R*_a_ value up to 99.2% after 24 h of incubation. In comparison, Huang *et al.*^18^ at 2017 developed a chitosan/gelatin coating incorporated with ZnO nanoparticles on pure Ti foil, and its mean antibacterial rate against planktonic *S. aureus* was up to 99.02% after 24 h incubation, chitosan/gelatin as the control. The result is very close to the TNTs/Ag + CS–Gel–Ag–ZnO coating in our study.

On the other hand, the antibacterial rate of TNTs/Ag did not show much increase (*R*_a_ = 37.4%) compared to the TNTs (*R*_a_ = 16.1%). With regard to the bactericidal capability of ZnO and CS as mentioned before, hence it is reasonable to assume that the reinforced antibacterial property of TNTs/Ag + CS–Gel–Ag–ZnO coating was attributed to the additional Ag, ZnO and CS contents in the CS–Gel–Ag–ZnO outlayer. The result is coincident with that of agar diffusion test, and provides evidence for the synergistic effect between ZnO and Ag.

In addition, the antibacterial rate of the control without TNTs/Ag (Ti + CS–Gel–Ag–ZnO) is up to 78.3%, which is lower than that of the TNTs/Ag + CS–Gel–Ag–ZnO (99.2%). Therefore, the TNTs/Ag layer combined with the CS–Gel–Ag–ZnO coating can be used for further enhancing the antibacterial rate. The sample without Ag (Ti + CS–Gel–ZnO) shows an antibacterial rate value of 42.7%, which is lower than that of Ti + CS–Gel–Ag–ZnO (78.3%), indicating that the Ag content in the CS greatly enhances the antibacterial ability of the coating. Besides, compared with Ti + CS–Gel–Ag–ZnO (78.3%), the Ti + CS–Gel–Ag sample has a lower antibacterial rate of 54.1%, indicating that the ZnO content in the CS also effectively improves the antibacterial ability of the coating. The Ti + CS–Gel sample still shows an antibacterial rate value of 22.1%, indicating that CS has certain ability of antibacterial activity. Furthermore, compared with Ti + CS–Gel, the antibacterial rate of Ti + CS–Gel–Ag increases, suggesting that both CS and Ag in the CS play important roles in killing planktonic bacteria.

According to Wang *et al.*,^42^ the antibacterial mechanism of Ag NPs embedded in TNTs can be divided into two modes: contact mode and non-contact mode. In the contact mode, the Ag NPs contact with bacteria and cause a series of lethal reactions. One of the most popular configurations for this process is the interaction between Ag NPs and the bacterial cell walls and membranes, which induces structural changes in the cell walls and membranes, increasing the permeability, and eventually leads to the death of the bacterium.^24^ Also, metallic silver is known to be reactive with sulfur and phosphorus containing compounds.^26^ The reactivity of Ag NPs is even higher due to the extremely high specific surface area. Therefore, the Ag NPs could also kill bacteria *via* binding to the thiol group of respiratory enzymes which inhibits respiratory process or penetrating inside the cell membrane where there are sulfur-containing proteins and phosphorus-containing DNAs.^22,25^ The SEM micrographs (Fig. 2) show randomly distributed Ag NPs (metallic, so as Fig. 6c indicates) adherent to the inner wall and top of TNTs. According to the agar diffusion test of TNTs/Ag coating (Fig. 8b) and its SEM micrographs (Fig. 9), there is no significant ZOI and no undistorted bacteria adhesion on the surface. Therefore, the Ag NPs in the TNTs/Ag sample should mainly undergo the contact-sterilizing mechanism. In the non-contact mode, the Ag NPs kill bacteria through releasing silver ions (Ag^+^) that dissolve into the surrounding fluid and interact with bacteria. However, the antibacterial rate of TNTs/Ag did not exhibit significant increase compared with TNTs, which indicates a low release rate of Ag^+^ of TNTs/Ag. Hence the burst release of Ag toward ambient environment is controlled. The TNTs/Ag is of low cytotoxic and shall not cause severe damages to the surrounding cells.

The antibacterial rate of TNTs/Ag + CS–Gel–Ag–ZnO is significantly higher than the TNTs/Ag and TNTs, due to the additional CS–Gel–Ag–ZnO outer layer. Chitosan alone is a strong bactericide, which releases amino ions (NH_2_)^+^ that could interact to the cell wall and increase its permeability. Gelatin alone do not have efficient antibacterial properties, but it could increase the viscosity of the CS matrix and help the ZnO and Ag NPs uniformly mixed. ZnO is also a strong bactericide. Besides, it is important to note that ZnO and Ag NPs could mutually enhance their antibacterial efficacy following a synergistic mechanism.^35,41,43,44^ Jin *et al.*^19^ co-implanted Zn and Ag into Ti *via* plasma immersion ion implantation (PIII). The antibacterial rates of Zn/Ag co-implanted Ti against both *E. coli* and *S. aureus* were higher than those of Zn or Ag implanted Ti. Acting as the host of Ag NPs, ZnO prevents Ag NPs from aggregation and thus increases the relative exposure area for contact-sterilization. Also, ZnO as an electron accepter reduces the charge density of Ag NPs, as is validated by XPS data (Fig. 6). The antibacterial activity is enhanced by strong electrostatic forces between positively charged Ag NPs and negatively charged bacteria. On the other hand, the increase of antibacterial activity does not necessarily lead to strong cytotoxicity. Since the many amide and hydroxyl groups in the chitosan molecule produce an affinity for metal ions such as Ag^+^, which reduces cytotoxic metal ions to metallic state, the cytotoxicity of TNTs/Ag + CS–Gel–Ag–ZnO coating is controlled.

## 4. Conclusions

Using pure titanium as a substrate, we obtained a TNTs/Ag + CS–Gel–Ag–ZnO coating with excellent antibacterial properties by anodization and electrodeposition. The results of SEM and other characterization method indicate that the Ag NPs firmly adhered to and were evenly distributed in the TNTs, and the Ag^+^ release rate was significantly reduced, which should be helpful for reducing the cytotoxicity and prolonging its life-span. The introduction of the CS–Gel–Ag–ZnO layer greatly improved the antibacterial properties of the surface. The cross-cut tape test showed that the coating adhered firmly to the substrate probably due to the large interface area between TNTs and CS–Gel. In subsequent microbiological tests, we compared the antibacterial properties of three systems: TNTs, TNTs/Ag, and TNTs/Ag + CS–Gel–Ag–ZnO. The results show that the Ag NPs in TNTs were very effective against adherent bacteria, but their ability to inhibit planktonic bacteria was very limited, while the CS–Gel–Ag–ZnO layer made up for this: The antibacterial rate reached a satisfactory 99.2%, thanks to the intrinsic antibacterial activity against planktonic bacteria of the CS, ZnO and Ag NPs, and the synergistic effect between ZnO and Ag. We expect that the antibacterial properties provided by this novel coating are sufficient for the application of titanium in orthopedic and dental implants.

## Conflicts of interest

There are no conflicts to declare.

## Supplementary Material

## References

[cit1] Surmeneva M. A., Vladescu A., Surmenev R. A., Pantilimon C. M., Braic M., Cotrut C. M. (2016). RSC Adv..

[cit2] Dai X., Zhang X., Xu M., Huang Y., Heng B. C., Mo X., Liu Y., Wei D., Zhou Y., Wei Y., Deng X., Deng X. (2016). RSC Adv..

[cit3] Simka W., Krząkała A., Masełbas M., Dercz G., Szade J., Winiarski A., Michalska J. (2013). RSC Adv..

[cit4] Geetha M., Singh A. K., Asokamani R., Gogia A. K. (2009). Prog. Mater. Sci..

[cit5] Brånemark P. I., Hansson B. O., Adell R., Breine U., Lindström J., Hallén O., Ohman A. (1977). Scand. J. Plast. Reconstr. Surg. Suppl..

[cit6] Van Noort R. (1987). J. Mater. Sci..

[cit7] Long M., Rack H. J. (1998). Biomaterials.

[cit8] Prabu V., Karthick P., Rajendran A., Natarajan D., Kiran M. S., Pattanayak D. K. (2015). RSC Adv..

[cit9] Wang Z., Xing M., Ojo O. (2014). RSC Adv..

[cit10] Niinomi M., Nakai M., Hieda J. (2012). Acta Biomater..

[cit11] Albrektsson T., Brånemark P. I., Hansson H. A., Lindström J. (1981). Acta Orthop. Scand..

[cit12] Di Martino A., Sittinger M., Risbud M. V. (2005). Biomaterials.

[cit13] Le Guéhennec L., Soueidan A., Layrolle P., Amouriq Y. (2007). Dent. Mater..

[cit14] Ma K., Cai X., Zhou Y., Wang Y., Jiang T. (2017). Macromol. Biosci..

[cit15] Li P., Zhang X., Xu R., Wang W., Liu X., Yeung K. W. K., Chu P. K. (2013). Surf. Coat. Technol..

[cit16] Nie B. e., Long T., Li H., Wang X., Yue B. (2017). RSC Adv..

[cit17] Hunagund S. M., Desai V. R., Kadadevarmath J. S., Barretto D. A., Vootla S., Sidarai A. H. (2016). RSC Adv..

[cit18] Huang P., Ma K., Cai X., Huang D., Yang X., Ran J., Wang F., Jiang T. (2017). Colloids Surf., B.

[cit19] Jin G., Qin H., Cao H., Qian S., Zhao Y., Peng X., Zhang X., Liu X., Chu P. K. (2014). Biomaterials.

[cit20] Lingzhou Z., Paul K. C., Yumei Z., Zhifen W. (2009). J. Biomed. Mater. Res., Part B.

[cit21] Regiel A., Irusta S., Kyziol A., Arruebo M., Santamaria J. (2013). Nanotechnology.

[cit22] Feng Q. L., Wu J., Chen G. Q., Cui F. Z., Kim T. N., Kim J. O. (2000). J. Biomed. Mater. Res..

[cit23] Kahru A., Dubourguier H. C. (2010). Toxicology.

[cit24] Kim J. S., Kuk E., Yu K. N., Kim J. H., Park S. J., Lee H. J., Kim S. H., Park Y. K., Park Y. H., Hwang C. Y., Kim Y. K., Lee Y. S., Jeong D. H., Cho M. H. (2007). Nanomedicine.

[cit25] Morones J. R., Elechiguerra J. L., Camacho A., Holt K., Kouri J. B., Ramírez J. T., Yacaman M. J. (2005). Nanotechnology.

[cit26] Rai M., Yadav A., Gade A. (2009). Biotechnol. Adv..

[cit27] Wan X., Wu L., Pei H., Ke H., Yang G., Tang J. (2018). RSC Adv..

[cit28] Ferraris S., Spriano S. (2016). Mater. Sci. Eng., C.

[cit29] Liu J., Liu B., Ni Z., Deng Y., Zhong C., Hu W. (2014). Electrochim. Acta.

[cit30] Luo H., Xiong P., Xie J., Yang Z., Huang Y., Hu J., Wan Y., Xu Y. (2018). Adv. Funct. Mater..

[cit31] Wan Y., Yang Z., Xiong G., Guo R., Liu Z., Luo H. (2015). J. Power Sources.

[cit32] Amin Yavari S., Loozen L., Paganelli F. L., Bakhshandeh S., Lietaert K., Groot J. A., Fluit A. C., Boel C. H. E., Alblas J., Vogely H. C., Weinans H., Zadpoor A. A. (2016). ACS Appl. Mater. Interfaces.

[cit33] Zhao C., Feng B., Li Y., Tan J., Lu X., Weng J. (2013). Appl. Surf. Sci..

[cit34] Porchezhiyan V., Noorjahan S. E. (2016). RSC Adv..

[cit35] Ghosh S., Goudar V. S., Padmalekha K. G., Bhat S. V., Indi S. S., Vasan H. N. (2012). RSC Adv..

[cit36] Liu Y., Kim H. I. (2012). Carbohydr. Polym..

[cit37] Xie K., Sun L., Wang C., Lai Y., Wang M., Chen H., Lin C. (2010). Electrochim. Acta.

[cit38] Zhang S., Peng F., Wang H., Yu H., Zhang S., Yang J., Zhao H. (2011). Catal. Commun..

[cit39] Ro C. U., Osán J., Van G. R. (1999). Anal. Chem..

[cit40] Szalóki I., Osán J., Worobiec A., de Hoog J., Van Grieken R. (2001). X-Ray Spectrom..

[cit41] Chen S., Wu G., Zeng H. (2005). Carbohydr. Polym..

[cit42] Wang J., Li J., Qian S., Guo G., Wang Q., Tang J., Shen H., Liu X., Zhang X., Chu P. K. (2016). ACS Appl. Mater. Interfaces.

[cit43] Lu W., Liu G., Gao S., Xing S., Wang J. (2008). Nanotechnology.

[cit44] Matai I., Sachdev A., Dubey P., Uday Kumar S., Bhushan B., Gopinath P. (2014). Colloids Surf., B.

